# Optimization of Sulfonated Polycatechol:PEDOT Energy Storage Performance by the Morphology Control

**DOI:** 10.3390/nano12111917

**Published:** 2022-06-03

**Authors:** Anatoliy A. Vereshchagin, Vasiliy V. Potapenkov, Petr S. Vlasov, Daniil A. Lukyanov, Oleg V. Levin

**Affiliations:** Institute of Chemistry, Saint Petersburg University, 199034 St. Petersburg, Russia; anatoliy_ve@mail.ru (A.A.V.); bacuy.99@mail.ru (V.V.P.); petr_vlasov@mail.ru (P.S.V.); lda93@yandex.ru (D.A.L.)

**Keywords:** polythiophene, PEDOT, quinone, catechol, redox polymer, conductive polymer, rechargeable batteries

## Abstract

Anionic catechol-containing polymers represent a promising class of functional dopants for the capacity improvement of conductive polymers. For example, sulfonated poly(vinylcatechol) SPVC with outstanding theoretical capacity was used as a dopant for poly(ethylenedixythiophene) (PEDOT) conductive polymer, increasing its energy storage performance. However, such materials suffer from insufficient utilization of the theoretical capacity of SPVC originating from non-optimal morphology. In the present study, we performed systematic optimization of the composition and morphology of the PEDOT:SPVC material as a function of the deposition parameters to overcome this problem. As a result, a capacity of 95 mAh·g^−1^ was achieved in a thin film demonstrating considerable electrochemical stability: 75% capacity retention after 100 cycles and 57% after 1000 cycles. Since the capacity was found to suffer from thickness limitation, a nanocomposite of PEDOT:SPVC and single-walled carbon nanotubes with high PEDOT:SPVC loading was fabricated, yielding the capacitance 178 F·g^−1^ or 89 F·cm^−2^. The capacity values exceed non-optimized film twofold for thin film and 1.33 times for nanocomposite with carbon nanotubes. The obtained results demonstrate the importance of fine-tuning of the composition and morphology of the PEDOT:SPVC materials to ensure optimal interactions between the redox/anionic and conductive components.

## 1. Introduction

Polycatechol redox polymers are of great interest as energy storage materials due to their outstanding theoretical energy density in combination with ecological and safety benefits [1]. Various synthetic [2,3,4] and bioinspired polycatechols [5,6,7,8,9] are implemented as cathode materials for different types of rechargeable batteries and supercapacitors. Due to the low conductivity of pristine polycatechols, the effective utilization of their energy requires wiring between their redox groups and the electrode surface, which can be achieved by inclusion in the matrix of conductive polymer (CP) [10,11,12].

Energy storage performance of such interpenetrating network materials strongly depends on their morphology and phase composition. Simple interchain entanglement of the components is insufficient to form stable and homogeneous material, which results in significant phase separation when the polycatechol forms domains disconnected from the CP network. The phase homogeneity of the interpolymer material can be increased by exploiting the ability of CPs to attain positive charge upon oxidation. The latter offers the possibility of introducing polyanions as the dopants during the oxidative synthesis of CPs, which is widely used in PEDOT:PSS and related materials [13]. Coulombic attraction between the positively charged CP chains and negatively charged polyanions increases the homogeneity and dissolution stability of the material.

**Figure 1:**
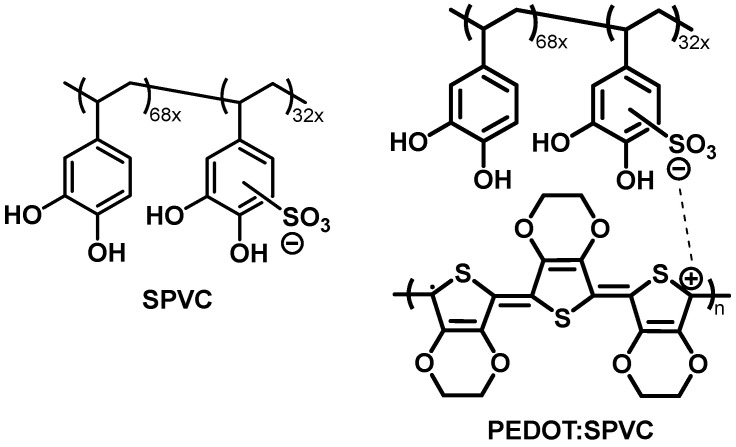
Chemical structure of the SPVC polyanion previously developed by our group and schematic representation of the PEDOT:SPVC material.

In the case of redox-active polyanions, this approach provides closer contact of the redox fragments with the conductive CP matrix, affording CP:polycatechol materials with good energy storage properties [14,15,16,17]. For example, CPs doped with sulfonated lignin showed capacity values up to 73 mAh·g^−1^ [18,19,20]. A more capacitive replacement for the sulfonated lignin was proposed on the basis of synthetic anionic polycatechols with theoretical capacities exceeding 250 mAh·g^−1^ [6,21]. Recently, we have reported an example of such a polymer, a sulfonated polycatechol (SPVC, Figure 1) with reduced molar mass of the monomeric unit (optimal design), which delivered improved theoretical capacity up to 330 mAh·g^−1^ [22]. However, practical capacity of these materials appears to be significantly lower than the theoretical value. Mainly, this is due to the low electrochemical availability of the catechol component caused by phase inhomogeneity, highlighting the importance of morphology optimization to improve the energy storage performance of the interpolymeric materials.

The main goal of the present work was to reveal the factors influencing the electrochemical availability of the anionic dopant and to improve the use of the theoretical capacity of the poly(3,4-ethylenedioxythiophene) (PEDOT) doped with the anionic polycatechol SPVC (PEDOT:SPVC, Figure 1). We have investigated the effect of the electrochemical deposition conditions on the performance of the resulting materials. The capacity of the polymer was found to be strongly dependent on the ionic and solvent composition of the deposition solution, electrodeposition mode and substrate morphology.

## 2. Materials and Methods

**General methods.** CHN(S) elemental analysis was performed using LECO TruSpec MICRO analyzer (Leco Instrumente GmbH, Mönchengladbach, Germany). Poly(3,4-dimethoxy)styrene with *M*_w_ = 130 kDa and Đ = 1.64 [4], poly(3,4-dihydroxy)styrene and NaSPVC [22] were obtained as described previously. SEM images were obtained on a Zeiss Merlin microscope (Carl Zeiss AG, Jena, Germany). XPS spectra of the studied samples were recorded using a Thermo Fisher Scientific Escalab 250Xi spectrometer (Thermo Fisher Scientific, Inc., Loughborough, UK) with non-monochromatic AlKα radiation (photon energy 1486.6 eV). Total energy resolution of the experiment was about 0.3 eV. Spectra of the samples were recorded in the constant pass energy mode at 20 eV, using a 650 mm diameter analysis area at room temperature in an ultrahigh vacuum of the order of 10^−9^ mbar. The ratio of PEDOT to SPVC was calculated using the sums of XPS signals area of 2p^3/2^ and 2p^1/2^ sulfur electrons., i.e., the ratio of signals areas related to PEDOT and SPVC: [(PEDOT)2p^3/2^ + 2p^1/2^]/[(SPVC)2p^3/2^ + 2p^1/2^]. To calculate the SPVC:PEDOT ratio, the SPVC fraction value was divided by the sulfonation degree of SPVC (0.32), which was determined by CHN(S) elemental analysis.

**Electrochemistry.** Electrochemical experiments were carried out on Biologic VMP3 multipotentiostat (BioLogic Sciences Instruments, Seyssinet-Pariset, France) or Autolab PGStat 302N (Metrohm AG, Herisau, Switzerland) in standard three-electrode cells in 0.1 M aqueous HClO_4_ electrolyte (unless otherwise specified). All potentials are referenced to silver chloride electrode (Ag|AgCl, NaCl_sat_). All capacity values presented in the paper were normalized to the active material mass. Glassy carbon (GC) electrode (0.07 cm^2^) was prepared by being polished with diamond powder, rinsed with ethanol and air-dried prior to experiments. Platinum electrode (11.4 cm^2^) was prepared by washing with acetone, water, conc. HCl and flame cleansing.

CNT felt was prepared by vacuum filtration of the 0.1% suspension of single-wall carbon nanotubes in toluene through the 0.45 µm PTFE membrane and subsequent drying at 60 °C for 24 h. PStat deposition was carried out using potentiostatic mode at 1.1 V potential until the current plateau was reached (4–10 min with intermittent washing and drying). Pulse deposition was performed using 1500 square 1:2 pulses of 2 s each. Both depositions were performed in a flow cell with continuous electrolyte filtration through the working electrode with 10 mL min^−1^ flow rate to facilitate the convection.

**IDE conductance.** *Operando* conductance measurements were performed on the IDE electrode as described elsewhere [23,24]. The film was deposited to the constant conductance from an optimal deposition electrolyte. The electrode was washed with H_2_O, and then its conductance was determined in 0.1 M HClO_4_/H_2_O. Cyclic voltammetry at 5 mV·s^−1^ was performed simultaneously on the IDE grids as two working electrodes with a constant difference of 10 mV between them. Currents, passing through the working electrodes, include the Faraday currents of the electrochemical process and the leakage ohmic current. If we assume that the magnitude of the Faraday currents is the same on both working electrodes, it is quite easy to determine the leakage current, and, according to Ohm’s law, determine the polymer resistance (or conductance). During the potential scanning, the current flowing through the working electrodes WE–1 and WE–2 involves the faradaic current of the electrochemical process *I*_F_ and the leakage current *I*. If we assume that the faradaic currents are equal, then we can write that:

(1) $$I_{WE-1}=I_{F}-I$$

(2) $$I_{WE-1}=I_{F}+I$$

and thus

(3) $$I=\frac{I_{WE-1}-I_{WE-2}}{2}$$

Given the leakage current and the bias between the combs, we can calculate the conductance (*G*) or resistance (*R*) of the polymer layer:

(4) $$G=\frac{1}{R}=\frac{I}{2v}$$

where Δ*I* is the difference between the currents on working electrodes and *V* is the potential difference between the working electrodes.

**EQCM.** EQCM experiments were performed on Biologic VMP3 multipotentiostat coupled with SRS QCM200 module (Stanford Research Systems, Sunnyvale, CA, USA) on quartz piezoelectric crystals coated with Pt (1.37 cm^2^, 5 MHz crystal frequency). The quartz resonance frequency was registered with a 200 Hz V^−1^ resolution during whole CV tests. *Operando* tests were performed in the standard EQCM compartment using the three-electrode scheme with Pt counter electrode and Ag|AgCl, NaCl_sat_, reference electrode in 0.1 M HClO_4_ at (−0.4 ÷ 1.2) V for deposition experiment and (−0.2 ÷ 0.8) V for ion flux determination at 50 mV s^−1^ scan rate. PEDOT:SPVC film was deposited on EQCM crystal from 10 mM EDOT + 10 mM NaSPVC or 20 mM NaPSS, respectively.

The mass of the film on the electrode surface was calculated as reported previously [23] according to the Sauerbrey equation [25]. The Sauerbrey equation in this form is only applicable for thin rigid films, i.e., when the product of immobilized film viscosity and density does not change significantly throughout the experiment [26]. To verify whether the crystal complies with the condition of a thin film, the viscoelastic phenomena were checked by monitoring changes of the crystal motional resistance.

**UV-Vis spectroelectrochemistry.** UV-Vis spectroelectrochemical experiments were performed using Autolab PGStat 302N and modular Avantes spectrophotometric system (Avantes B.V., Apeldoorn, The Netherlands). Experiments were carried out in the three-electrode cell made from 1 cm quartz cuvette with transparent ITO/glass electrodes of 8 mm width, Pt counter electrode and Ag|AgCl, NaCl_sat_, reference electrode in 0.1 M HClO_4_ at (−0.4 ÷ 1.2) V for deposition experiment and (−0.2 ÷ 0.8) V for spectroelectrochemical experiments at 50 mV·s^−1^ scan rate. PEDOT:SPVC film was deposited on ITO electrode from 10 mM EDOT + 10 mM NaSPVC or 20 mM NaPSS, respectively. Sampling interval of the spectrometer was 20 s.

## 3. Results

### 3.1. Optimization of the Electrochemical Deposition

Films of PEDOT:SPVC were obtained by the oxidative electrochemical co-deposition of the EDOT in the presence of NaSPVC as a supporting electrolyte. During the oxidative polymerization, PEDOT chains attain some positive charge compensated for by negatively charged SPVC chains, which results in interpolymeric material composed of interpenetrating PEDOT and SPVC chains held together by Coulombic forces. According to the literature, electrochemical properties of the electrodeposited PEDOT films doped with polyanions are strongly affected by synthesis conditions such as solvent, reagent ratio, additional supporting electrolyte, deposition mode, etc. To find the optimal conditions for the electrodeposition of PEDOT:SPVC, we studied how the electrochemical response of the deposited film depends on the reaction parameters. According to the previous literature reports [21] and our findings [22], polycatechols tend to form isolated domains which are not penetrated by CP chains within the interpolymer materials. As we have found, the material deposited by using the method established for the PEDOT:PSS contained only a small fraction of the PEDOT, which may lead to insufficient interpenetration with the SPVC and thus low electrochemical availability of SPVC.

**Figure 2:**
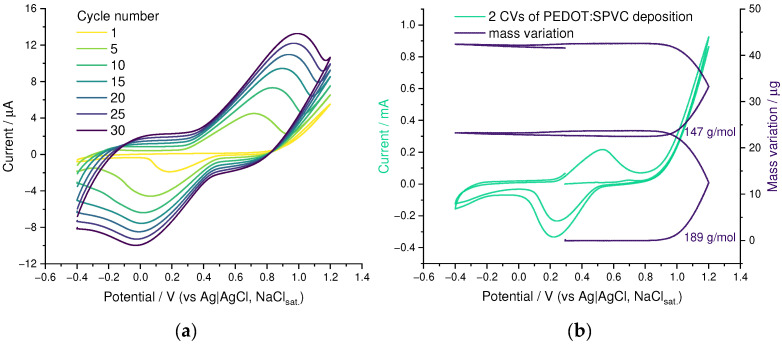
(**a**) CV and (**b**) EQCM curves for electrochemical oxidation of 10 mM EDOT + 10 mM NaSPVC, 20 mV·s^−1^, 20% CH_3_CN in H_2_O.

On a CV of EDOT solution containing NaSPVC, cycle-to-cycle growth of both catechol redox peak pair at 0.6 V and 0.2 V and broad rectangular-shaped capacitive response in the whole CV range, originating from the PEDOT, were observed, indicating PEDOT:SPVC film growth on electrode surface (Figure 2a). According to EQCM, polymerization occurred at potentials above 0.9 V, corresponding to an onset of the EDOT oxidation peak (Figure 2b). The mass flux during the deposition comprised 189 G·mol^−1^, which corresponds to 0.5 EDOT molecule + ca. 0.8 monomeric unit of SPVC.

According to the published data [21], insufficient fraction of the PEDOT in the composite materials with anionic redox polymers may negatively affect the electrochemical performance of the material due to the poor density of the PEDOT charge-transport channels and thus low electrochemical availability of the redox polymer. We have examined two approaches for increasing the PEDOT fraction in the PEDOT:SPVC material.

**Figure 3:**
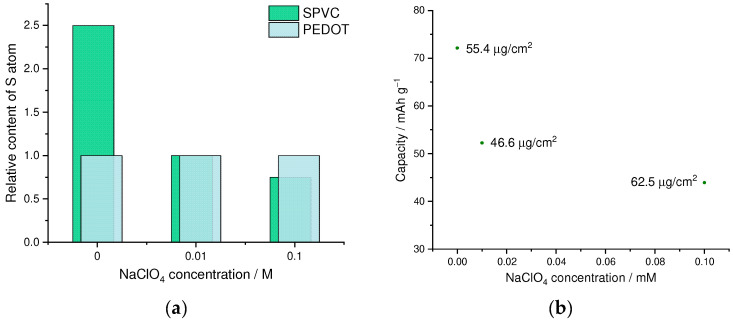

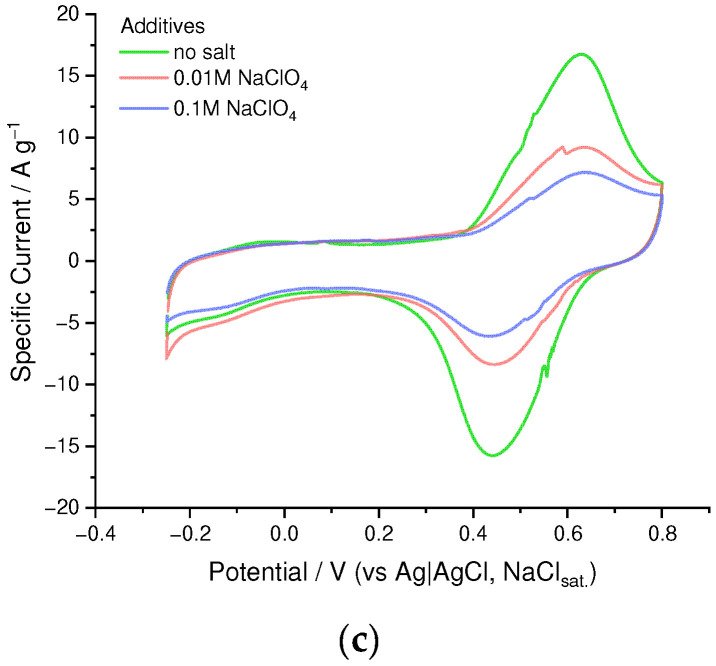
(**a**) SPVC to PEDOT ratio by XPS, (**b**) gravimetric capacity vs. NaClO_4_ concentration and (**c**) CV curves (20 Mv·s^−1^, 0.1 mM HClO_4_/H_2_O) for PEDOT:SPVC films deposited with different NaClO_4_ additives; deposition from 5 mM EDOT + 10 mM NaSPVC, 10% CH_3_CN in H_2_O.

First of all, we have studied the effect of addition of the low-molecular anionic dopant on the PEDOT to SPVC ratio and electrochemical performance of the material. We performed a series of competitive doping experiments with different additives of ClO_4_^−^ as a low-molecular anionic dopant. With increasing perchlorate concentration, a fraction of SPVC in the material dropped, which was clearly visible on a CV and was confirmed by XPS analysis (Figure 3a, and Figures S1–S4). In the absence of perchlorate, molar ratio of the SPVC to PEDOT sulfur atoms comprised 2.5:1, which was a largely excessive anionic doping of the PEDOT. Considering the sulfonation degree of SPVC (0.32), this material contained ca. 7.8 catechol units per one monomeric unit of PEDOT. Addition of 0.01 and 0.1 M NaClO_4_ decreased the catechol to PEDOT ratio to 3.1:1 and 2.3:1, respectively. As a result, capacity values turned out to be significantly lower with increasing perchlorate concentration (Figure 3b). The catechol redox peak currents were significantly decreased by addition of NaClO_4_, while the PEDOT currents stayed at the same value due to the decreasing SPVC content (Figure 3c). Such capacity decrease cannot be explained directly by insufficient SPVC content in the material, since at 2.3:1 component ratio the theoretical capacity was still far above experimental values, which means that the content of electrochemically available SPVC changed with the whole SPVC content in the material.

**Figure 4:**
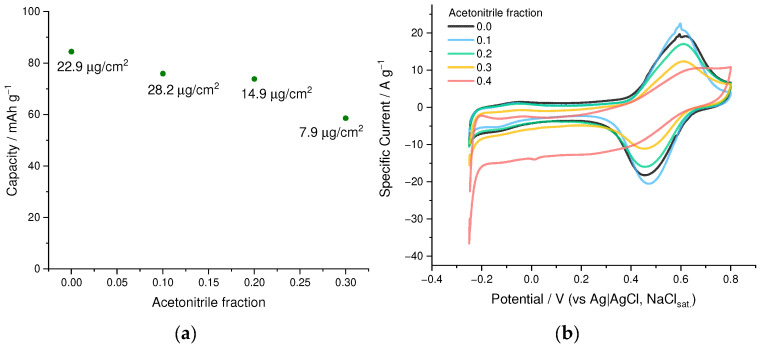
(**a**) Gravimetric capacity and areal loading vs. CH_3_CN fraction in the deposition electrolyte and (**b**) CV curves (20 mV·s^−1^, 0.1 mM HClO_4_/H_2_O) of PEDOT:SPVC films; deposited from 5 mM EDOT + 10 mM NaSPVC solutions with different CH_3_CN fraction.

Polymerization of EDOT is typically carried out from the organic or water-organic solvent mixtures or using the surfactants [27] due to the limited solubility in water, while NaSPVC polymer is highly hydrophilic and does not dissolve in most organic solvents. To find out the optimal balance for the dissolution of both components of the deposition electrolyte, we examined the influence of the H_2_O/CH_3_CN ratio on the capacity of electrodeposited PEDOT:SPVC films (Figure 4a). Although the EDOT solubility in pure water is ca. 15 mM [28], the addition of NaSPVC to such a solution causes separation of the EDOT phase from 5–10 mM solutions, which leads to uncertainties about the effective EDOT concentration in the solution. This problem can be solved by addition of 20% of CH_3_CN. At higher acetonitrile content, the charge of the catechol peak decreased sharply, and at 40% of acetonitrile, it almost disappeared (Figure 4b). This may be explained by the transition of SPVC chains in solution from elongated to globular conformation caused by the increase of the acetonitrile fraction. Indicative of this is a slight opaqueness and lower viscosity of the solution in 40% acetonitrile. As a result, 20% acetonitrile in water was established as an optimal solvent system for the deposition of PEDOT:SPVC films.

**Figure 5:**
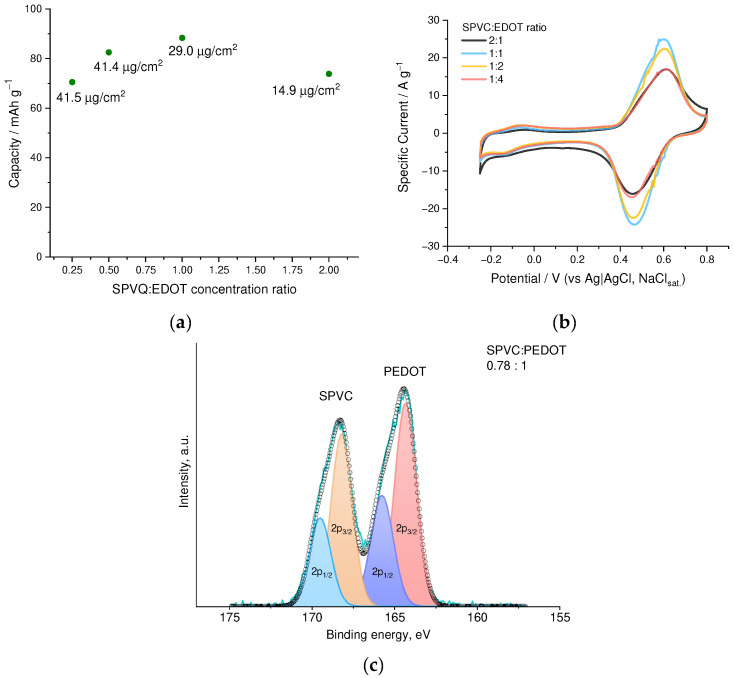
(**a**) Gravimetric capacity and areal loading vs. NaSPVC:EDOT molar ratio in the deposition electrolyte, (**b**) CV curves (20 mV·s^−1^, 0.1 mM HClO_4_/H_2_O) of PEDOT:SPVC films deposited from EDOT + NaSPVC solutions with different molar ratio and (**c**) fitted XPS spectrum of the PEDOT:SPVC deposited from 1:1 solution; deposition from 20% CH_3_CN in H_2_O.

Alternatively, composition and, thus, electrochemical properties of the deposited films may be tuned by variation of SPVC:EDOT molar ratio in the electrolyte. A series of films was deposited from electrolytes with SPVC:EDOT molar ratio ranging from 1:4 to 2:1 (Figure 5a,b). As a result, the film deposited from 1:1 solution of SPVC:EDOT showed the best capacity value of 88.4 mAh·g^−1^. The SPVC to PEDOT molar ratio in this film comprises 2.4:1 according to XPS (Figure 5c). Practical capacity values, calculated by subtraction of the PEDOT:PSS baseline from the CV of the PEDOT:SPVC, comprised 117 mAh·g^−1^ or 35% of the theoretical capacity of SPVC with given sulfonation degree.

**Figure 6:**
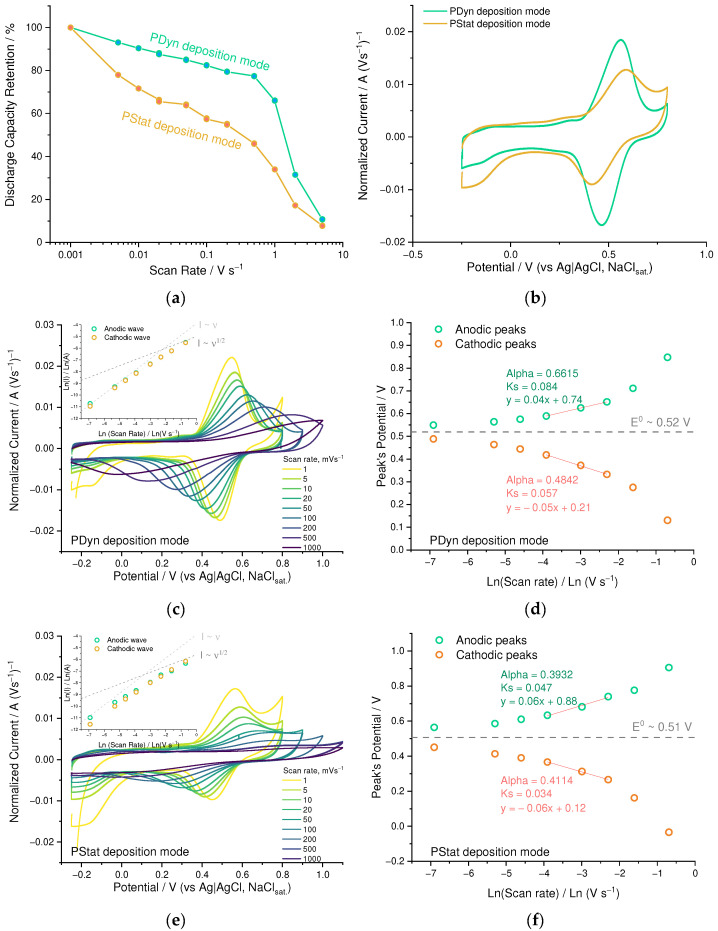
(**a**) Gravimetric capacity retention vs. scan rate and (**b**) CV curves (20 mV s^−1^, 0.1 mM HClO_4_/H_2_O) for Pdyn and Pstat films; (**c**,**e**) CV curves at different scan rates (0.1 mM HclO_4_/H_2_O) of Pdyn and Pstat films, respectively (inset: dependence of peak current vs. scan rate); (**d**,**f**) dependence of peak potential vs. scan rate and rate constants for Pdyn and Pstat films, respectively.

To examine the influence of the polymerization technique on the properties of the resulting material, we compared the PEDOT:SPVC films deposited potentiostatically (PStat) and potentiodynamically in CV mode (PDyn) to the same areal loading (73 µg·cm^−2^ and 76 µg·cm^−2^, respectively). Electrochemical properties of the resulting film were found to depend strongly on electrochemical deposition mode. On the one hand, PStat film showed slightly higher capacity at low scan rates (78 vs. 73 mAh·g^−1^ at 1 mV·s^−1^) compared to PDyn film of the same areal loading. On the other hand, even after a small rate increase from 1 to 5 mV·s^−1^, PDyn film retained more capacity (Figure 6a).

Higher peak separation (Figure 6b) indicates kinetic limitations of the charge transfer in Pstat film. The nearly first-order relation between the logarithm of peak current and the logarithm of scan rate (insets, Figure 6c,e) also confirms the kinetics to be an overall limiting factor for the redox process of both PEDOT:SPVC films. Heterogeneous rate constants of the Pdyn film calculated using the Laviron method (Figure 6d, *k*_s_ = 0.084 s^−1^ for anodic peak and 0.057 s^−1^ for cathodic peak) were almost twofold higher than for Pstat film (Figure 6f, *k*_s_ = 0.047 s^−1^ for anodic peak and 0.034 s^−1^ for cathodic peak), indicating that in the latter material the charge transport was more impeded [29].

This effect may be explained by insufficient electrochemical availability of the SPVC particles due to the formation of isolated domains. Due to poor electronic conductivity within these domains, elevation of the scan rate hinders their elaboration and turns them off from the charging/discharging of the film. This was clearly visible from the scan rate variation experiment: SPVC peaks literally disappeared from the CV curve of the Pstat film at 200 mV·s^−1^, while for the Pdyn film they were visible up to 1000 mV·s^−1^ (Figure 6c,e). Capacitive response of the PEDOT was broader for the Pstat film when compared with the Pdyn film, which was clearly visible in the low-potential region of the CV.

**Figure 7:**
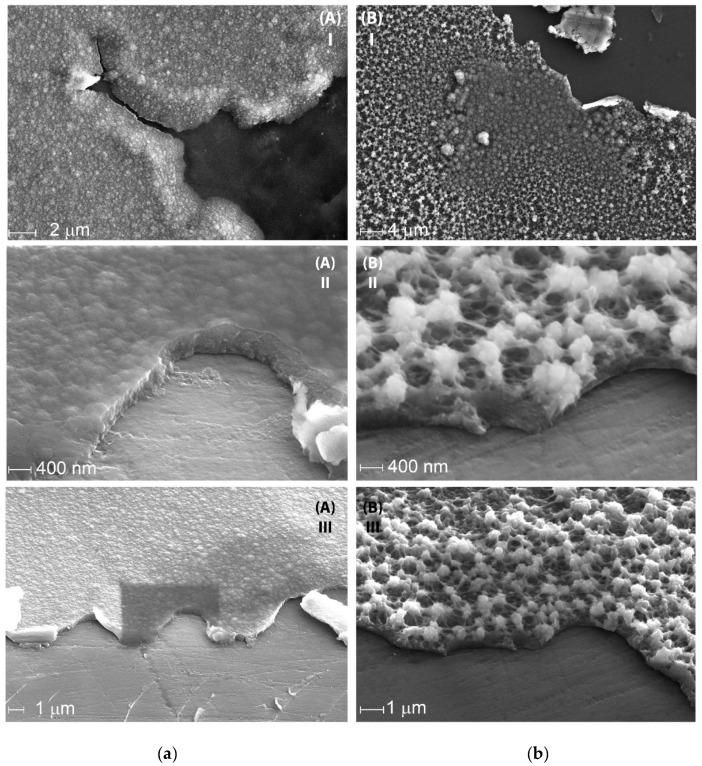

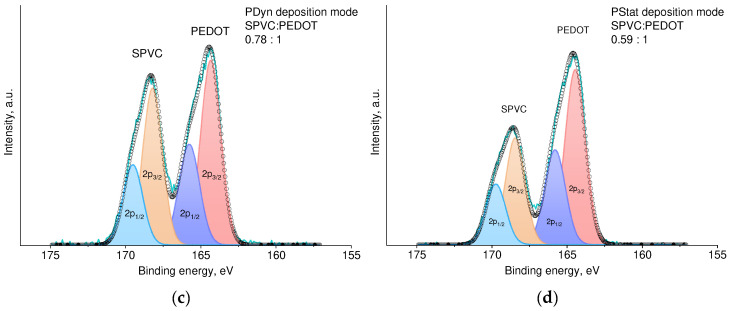
SEM images of (**a**) PDyn and (**b**) Pstat films at different magnifications; (**c**,**d**) fitted XPS spectra of Pdyn and Pstat films.

SEM images of the resulting films revealed significant morphology differences between these films (Figure 7a,b). While CV deposition results in a homogeneous film with a relatively flat surface, Pstat film shows a highly porous structure with distinct particles and clusters on the surface. The high brightness of these particles on SEM suggests that they are more conductive then the rest of the material and are likely enriched with the PEDOT component. This assumption was supported by the XPS spectroscopy data, showing that the Pstat sample contained more PEDOT compared with Pdyn (Figure 7c,d).

### 3.2. Ion Transport Studies

**Figure 8:**
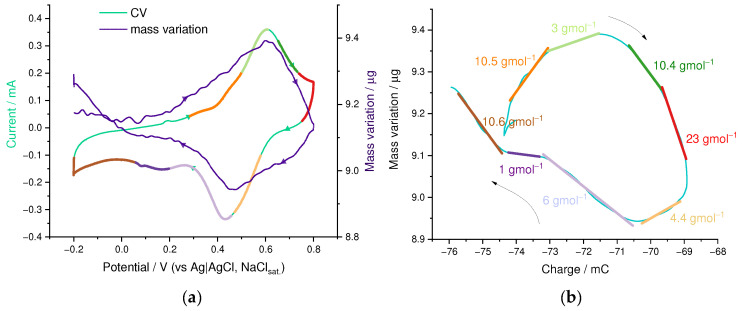
(**a**) Superimposed CV and Δ*m*(*E*) curves and (**b**) Δ*m*(*C*) curve with *m*/*z* slopes from EQCM experiment at 50th cycle, 0.1 M HclO_4_/H_2_O, 20 mV·s^−1^.

Ion transport in the PEDOT:SPVC film deposited on a platinum-coated EQCM electrode was studied in aqueous 0.1 M HclO_4_ electrolyte. The film was cycled until stabilization of the electrochemical and mass response before acquiring the EQCM curve. Mass of the film started to increase from ca. 0.2 V to 0.6 V, which corresponds to the oxidation peak potential of SPVC with an inbound flux with mean *m*/*z* value of 8 g·mol^−1^ (Figure 8). Since ClO_4_^−^ (*m*/*z* 99.5 g·mol^−1^) was the only anion present in the solution, the observed mixed transport consisted of the major outbound H^+^ flux superimposed with a minor inbound ClO_4_^−^ flux with transport numbers of 0.91 and 0.09, respectively. Above the catechol peak potential, an outbound flux of −10 g·mol^−1^ was observed, which can be described as a mixed H^+^–H_3_O^+^ transport.

On the backwards scan, an outbound flux of −4 g·mol^−1^ was detected, corresponding to an H^+^ inbound transport and a subtle fraction of opposite ClO_4_^−^ transport, with the transport numbers of 0.95 and 0.05, respectively. Below the peak potential of 0.46 V, ion transport switched to an inbound flux, consisting of three regions with *m*/*z* slopes of 6, 1 and 11 g·mol^−1^, which can be described by pure H^+^ inbound (for *m*/*z* = 1) or mixed H^+^–H_3_O^+^ inbound flux.

Summarizing this, the PEDOT:SPVC film showed primarily H^+^ transport, however, the ion fluxes were accompanied with water transport, namely shrinking upon oxidation and swelling upon reduction.

### 3.3. Thickness Limitation Studies

**Figure 9:**
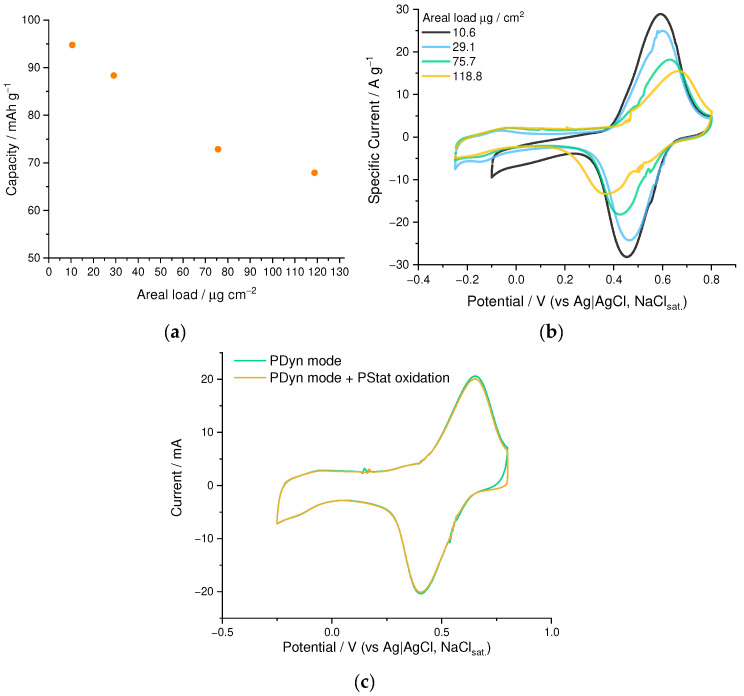
(**a**) gravimetric capacity vs. the areal loading and (**b**) CV curves of PEDOT:SPVC films deposited to different areal loadings; (**c**) CV of the PEDOT:SPVC film with and without potentiostatic oxidation at 0.8 V; 0.1 M HClO_4_/H_2_O, 20 mV·s^−1^.

We have found that the practical capacity of the PEDOT:SPVC films strongly depends on its thickness (Figure 9a,b). Practical gravimetric capacity of the PEDOT:SPVC film at 10.6 µg·cm^−2^ areal loading comprised 95 mAh·g^−1^, while increasing the areal loading lowered the capacity, affording only 67 mAh·g^−1^ at 118.8 µg·cm^−2^.

To exclude the diffusion limitations as the origin of the capacity decrease, we performed the oxidative saturation experiment during CV. While reaching an anodic boundary on the CV of PEDOT:SPVC film, we held the potential at this value until zero current was reached, meaning all processes including diffusion were brought to the quasi-equilibrium state. Subsequently recorded CV showed no difference from an ordinary CV of the same film, which indicates that the diffusion does not confine the degree of electrochemical conversion of the polymer, which allows us to exclude the diffusion limitations (Figure 9c).

This assumption was supported by comparison of the conductance of PEDOT:SPVC film with the model PEDOT:PSS using the difference voltammetry on a double-comb interdigitated electrode (IDE) using the protocol described previously [10,23]. Conductance of the PEDOT:SPVC film (Figure S10) was found to be one order of magnitude lower compared to the highly conductive reference PEDOT:PSS film on the same electrode (Figure S9).

### 3.4. Operando Spectroelectrochemical Studies

UV-Vis absorption spectroelectrochemistry (SEC) is widely used to monitor the redox transformation of CPs [30]. Identification of the polaronic charge carriers populating the CP backbone may be carried out by analyzing the evolution of the UV-Vis spectrum upon variation of the applied potential. We deposited a well-known PEDOT:PSS material using the same protocol as for production of PEDOT:SPVC to compare their behavior.

**Figure 10:**
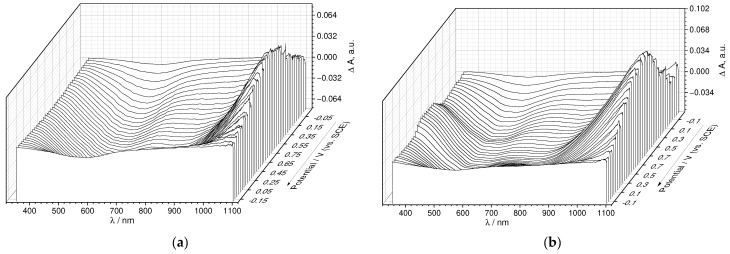

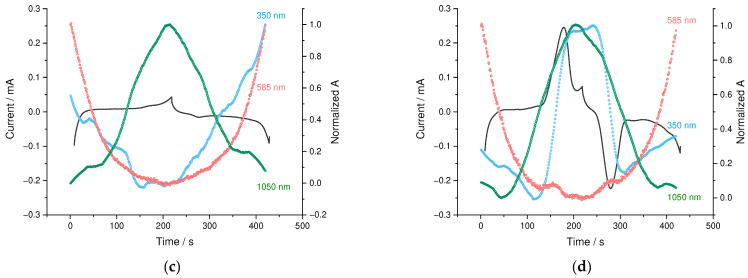
Three-dimensional spectroelectrochemical E-λ plots for (**a**) PEDOT:PSS and (**b**) PEDOT:SPVC films; CV and potential-dependent absorbance variations at selected wavelengths for (**c**) PEDOT:PSS and (**d**) PEDOT:SPVC films plotted vs. time; 0.1 M HClO_4_, 5 mV·s^−1^.

As seen from the differential 3D plots, composed of the UV-Vis spectra at certain points of the CV cycle (E-λ plots, Figure 10a,b, Figures S11 and Figure S12), both for PEDOT:PSS and PEDOT:SPVC, broad polaronic absorption emerged around 1050 nm upon oxidation, while absorption at 585 nm, attributed to the neutral PEDOT chains, decreased simultaneously [31]. These spectral changes were observed in the whole CV range, even at low potentials, which confirms their connection with the PEDOT-centered redox processes. Additionally, a new band around 380 nm with a shoulder to ca. 550 nm appeared in the spectra of PEDOT:SPVC recorded at potentials of the catechol redox peak and above. This band, originating from absorption of the *o*-quinone moiety, started to grow with the redox peak of SPVC. For both PEDOT:PSS and PEDOT:SPVC, observed spectral perturbations were reversible.

The PEDOT spectroelectrochemical response appeared to be nearly the same for both materials, which indicates that the SPVC component does not influence the redox processes on a PEDOT chain. To examine the charge distribution between the PEDOT and SPVC in the material, we performed an open circuit potential (OCP) relaxation spectroelectrochemical experiment. PEDOT:PSS and PEDOT:SPVC films were potentiostatically charged at 0.8 V until the zero current and then left in the electrolyte in OCP mode. UV-Vis spectra were recorded during the relaxation of the film to monitor the changes in the oxidation states of individual components.

**Figure 11:**
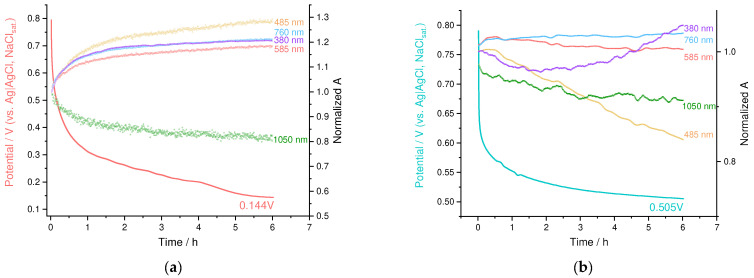
OCP and absorption relaxation plots for (**a**) PEDOT:PSS and (**b**) PEDOT:SPVC films; 0.1 M HClO_4_.

During the relaxation, PEDOT:PSS showed nearly exponential decay of the OCP value to 0.144 V after 6 h, which is indicative of slow discharge by contaminating electron donors from electrolytes (Figure 11a). Polaronic absorbance at 1050 nm decreased as well, retaining 0.8 of the initial absorbance, while the bands, corresponding to the reduced forms of PEDOT, increased proportionally.

OCP of the PEDOT:SPVC exhibited more complex behavior: initial potential drop from 0.8 to ca. 0.6 V occurred rapidly, followed by slower decay in the same manner with PEDOT:PSS (Figure 11b). The former process has quite a higher time constant and may be attributed to the intramolecular charge transfer. During the first hour of the experiment, the polaron band of PEDOT (1050 nm) decreased, while the intensity of the bands at 585 nm and 485 nm increased. Then, the second process started, which was found to be similar to the slow discharge by contaminating electron donors described above. Unlike PEDOT:PSS, slow discharge of PEDOT:SPVC was accompanied by decrease of the band at 485 nm, which to a large extent corresponds to the *o*-benzoquinine group [32]. This indicates the obvious fact that catechol fragments, which have higher redox potential, are reduced first.

### 3.5. Electrochemical Stability

**Figure 12:**
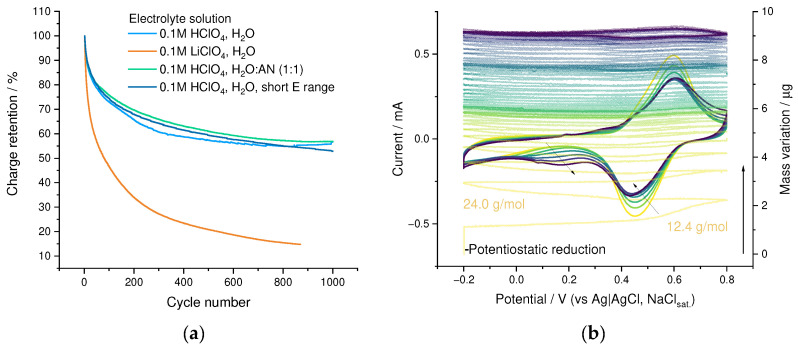

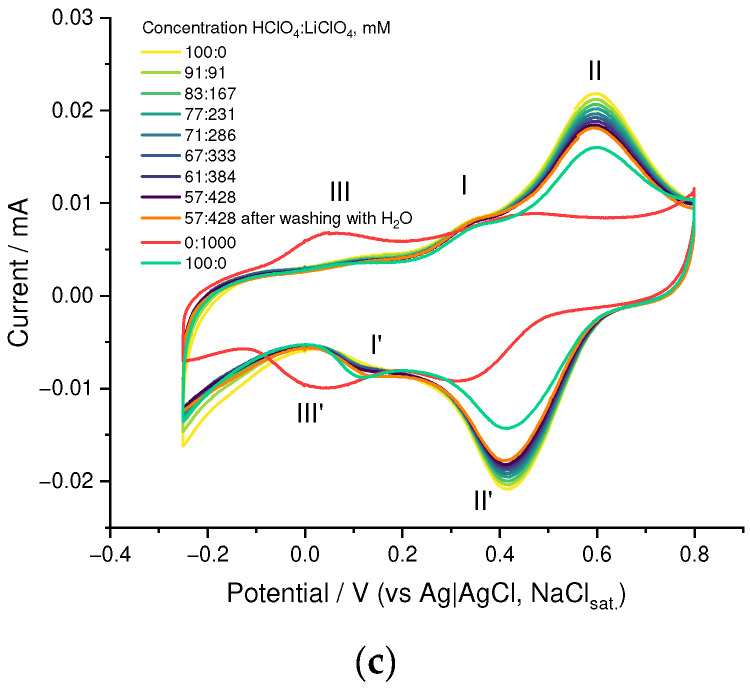
(**a**) Cycling stability of PEDOT:SPVC films in different cycling conditions, (**b**) 50 cycles of EQCM cycling of the PEDOT:SPVC film in 0.1 M HClO_4_, 20 mV·s^−1^ and (**c**) LiClO_4_ titration of the PEDOT:SPVC film.

The cycling stability of the resulting PEDOT:SPVC film was assessed during 1000 CV cycles in 0.1 M HclO_4_. The capacity retention comprised 75% after 100 cycles and 57% after 1000 cycles (Figure 12a). As seen from the multi-cycle EQCM experiment, the cycling of the PEDOT:SPVC film was accompanied by a substantial mass gain (ca. 22% of the initial film mass), diminishing from cycle to cycle (Figure 12b), indicating that the capacity loss is not linked with the dissolution or mechanical destruction of the film. On the first cycle, irreversible mass flux was 12 and 24 g mol^−1^ at the forward and backward CV branch, respectively, which was close to an irreversible inbound flux of H_2_O. The evolution of the CV curve shape clearly showed that the capacity loss was predominantly caused by the degradation of SPVC redox activity, while the rectangular capacitive signal of PEDOT remains constant (Figure 12b and Figure S5).

In addition to the degradation of the catechol electroactivity, a new redox process emerged at 0.3 V, which was not observed during the cycling of PEDOT-based materials, and thus may be explained by chemical transformation of SPVC upon repetitive oxidation in aqueous media. Two plausible pathways for such transformations can be suggested: C-C dimerization of the catechol unit or its hydroxylation. The current of this new observed peak pair grew mostly during the first 100 cycles. At the same time, no correlation was found between this process and the observed mass gain of the film, while the hydroxylation of SPVC should be accompanied with a continuous mass gain and redox peak growth until the complete utilization of the catechol fragments. On the contrary, the formation of C-C dimers does not change the film mass and, as a cross-linking process, is possible only for a limited fraction of the catechol units due to the decreasing flexibility of the polymer chains. Therefore, we conclude that the observed capacity degradation is most likely the result of C-C dimerization.

To discover optimal exploitation conditions for the obtained material, we performed a series of stability experiments. While cycling in short potential range (0.1–0.8 V), the capacity decay profile showed only a minor deviation from the one obtained in the broad cycling range (Figure 12a and Figure S6). A similar situation was observed when 0.1 M HClO_4_ in 1:1 water-acetonitrile was used as an electrolyte (Figure 12a and Figure S7). However, the CV and stability of the film changed dramatically when HClO_4_ electrolyte was replaced by LiClO_4_ (Figure 12a,c and Figure S8): a new peak pair, typical for catechols in Li^+^ electrolytes [33], emerged at 0.05 V, and the initial peak pair was shifted 0.15 V lower. The capacity degradation rate of PEDOT:SPVC film in LiClO_4_ electrolyte was more than twofold times higher compared with HClO_4_ electrolyte, and both peaks decreased simultaneously.

To examine the effect of Li^+^ addition on the electrochemical properties of the PEDOT:SPVC film in more detail, we performed an *operando* titration of the film, cycled in 0.1 M aqueous HClO_4_, with LiClO_4_ solution. The peak current drop upon addition of up to 0.75 M of LiClO_4_ lay within the normal cycling degradation extent of the PEDOT:SPVC. However, after the film was washed with water and immersed in 0.1 M LiClO_4_ electrolyte, the CV curve attained the two-peak form as described previously. Being placed again in the HClO_4_ electrolyte, the film restored the initial one-peak CV shape. Such electrochemical behavior of the film is typical for non-specific cationic transport: at low pH, proton transport absolutely prevails over lithium transport even at large excess of Li^+^ due to the extremely high proton mobility, but at neutral pH, the transport easily switches to Li^+^, while in acidic electrolyte the lithiated film easily restores the proton transport.

### 3.6. SWCNT-Supported PEDOT:SPVC

To overcome the observed thickness limitations of the capacity, we performed several attempts to deposit the PEDOT:SPVC material onto a felt electrode fabricated from single-walled carbon nanotubes (SWCNT). Due to the extremely high specific surface, SWCNT may allow high material loading and, at the same time, the layer thickness of the material coating lies below limitations. Combined with excellent electronic conductivity, the SWCNT seems to be an ideal substrate for the deposition of materials with strict thickness capacity limitations.

**Figure 13:**
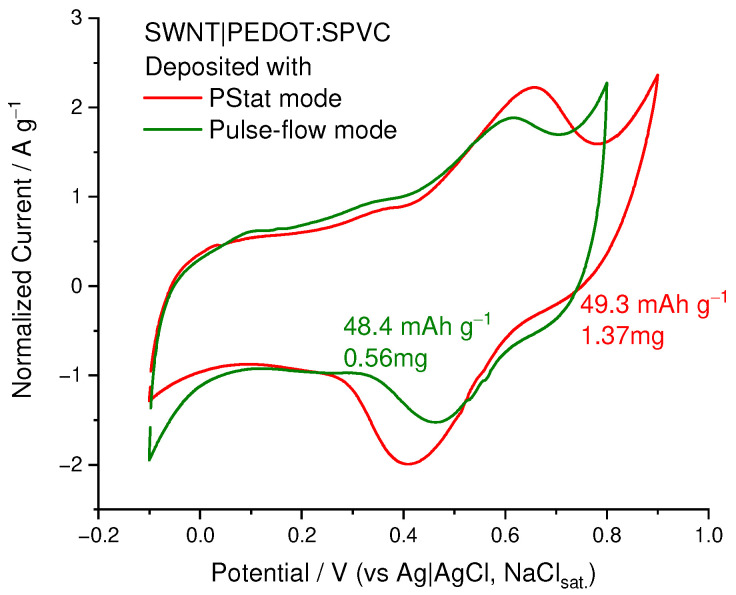
CV of the PEDOT:SPVC/SWCNT electrodes deposited using PStat and pulse modes, 20 mV·s^−1^, 0.1 M HClO_4_.

Deposition in CV mode, being the most suitable for plain electrodes, was found to be ineffective in the case of the SWCNT substrate with high surface area. First attempts at potentiostatic deposition performed in static electrolyte or upon conventional stirring were also unsuccessful, but the situation changed when we performed the continuous filtration of the electrolyte through the working electrode to facilitate the mass transfer inside the SWCNT felt. Potentiostatic deposition at 1.1 V in this regime afforded the PEDOT:SPVC/SWCNT nanocomposite electrode with high loading of an active material (1.37 mg at 2.7 mg of SWCNT). Capacity of the deposited material after subtraction of the background capacitance of SWCNT comprised 49.3 mAh g^−1^, which corresponds to the capacitance of 178 F·g^−1^ or 89 F·cm^−2^ (Figure 13).

As an alternative to the CV potentiodynamic deposition, pulse deposition was probed, which is known to be more suitable for the modification of carbon nanomaterials [34]. Deposition using 1:2 square pulses with 1.1 V amplitude resulted in formation of a 0.56 mg deposit on 3.9 mg of SWCNT with nearly the same resulting capacity (48.4 mAh·g^−1^, 174 F·g^−1^ or 86 F·cm^−2^). However, in this case, catechol peak currents were significantly lower.

## 4. Discussion

Despite a large number of studies devoted to the deposition of the conductive polymers doped with polyanions, e.g., the PEDOT:PSS, the described methods are mostly developed for the redox-neutral polyanions and thus are aimed at an improvement of the bulk conductivity of the materials rather than at the charge transfer between the components of the material. Optimization of the deposition conditions for the PEDOT:SPVC material performed reveals some trends of the relationship between the deposition conditions and electrochemical properties of the material. For example, PEDOT:SPVC electrodes with the same chemical composition but deposited from different electrolytes demonstrate unequal capacity (44 vs. 88.4 mAh·g^−1^) and CV behavior. This fact outlines the importance of the optimization of the PEDOT:SPVC material deposition to improve its energy storage performance.

According to the literature [21,22], insufficient electrochemical availability of the redox polymer is the main capacity-limiting factor in such materials as PEDOT:SPVC, so one of the main goals of the material design is to improve the density of the PEDOT interpenetrating network, which should be in close contact with any catechol unit of SPVC. Initially, we considered the addition of NaClO_4_ to increase the density of PEDOT network by raising the PEDOT fraction in the material, but as a result, an opposite effect on capacity was observed. As an explanation, we may propose that the PEDOT doped with low molecular perchlorate ion separates to a new phase, which impairs the interpenetration of the PEDOT and SPVC chains and the charge transfer between them. In summary, the deposition electrolyte should be free from low-molecular anions.

As an alternative, PEDOT content in a deposited material may also be affected by a composition of the deposition electrolyte. Since NaSPVC is the only ion source in the deposition solution, technical limitations such as electrolyte conductivity and EDOT solubility arise. This strongly confines the range of electrolyte compositions suitable for the electrodeposition of the PEDOT:SPVC material. Variation of the EDOT to SPVC ratio in electrolyte from 4 to 0.5 afforded a series of materials, the capacity of which showed peak-shaped dependence on an electrolyte composition with a maximum at 1:1 composition. Despite the increased electrochemical availability of SPVC at the higher PEDOT fraction, the loss of overall capacity due to the decreased SPVC fraction cannot be compensated. However, the difference between the best and the worst samples (88.4 vs. 70.3 mAh·g^−1^) was not so high compared with the NaClO_4_ effect.

Unfortunately, NaSPVC was found to decrease the solubility of EDOT in water, so we were forced to add 20% of CH_3_CN to the electrolyte to dissolve it completely. At the same time, CH_3_CN additive decreases the polarity of the media, enforcing the hydrophilic SPVC chains to attain more compact conformation [35], which prevents uniform distribution of relatively large SPVC macromolecules in the material. Although the capacity of the PEDOT:SPVC films obtained from electrolytes with CH_3_CN fraction below 20% was slightly higher, the electrolyte was obviously heterogeneous. Unfortunately, the conditions and the results of these experiments were not reproducible enough. In this case, an anionic surfactant may be used to stabilize the heterogeneous electrolyte while maintaining the SPVC in the elongated conformation, as described in literature [27].

Electrochemical deposition mode also affects the morphology and electrochemical properties of the PEDOT:SPVC film. Potentiodynamic deposition mode surpassed potentiostatic in this case, affording more homogeneous film with higher capacity and heterogeneous rate constant. As seen from the SEM images, PStat film was coated with the particles of the highly conductive phase, presumably pristine PEDOT. XPS confirmed that the PStat film contains more PEDOT then the PDyn film. This fact may be explained by a delayed diffusion of SPVC macromolecules. Due to this, the continuous depletion of the near-electrode layer with SPVC during potentiostatic deposition may lead to the formation of the PEDOT with low doping degree, while the CV allows the diffusion to restore SPVC concentration during the reduction part of the CV cycle.

After establishing optimal deposition conditions for the PEDOT:SPVC (potentiodynamic deposition from 10 mM EDOT + 10 mM NaSPVC solution in 20% CH_3_CN/H_2_O), we have focused on the factors determining the electrochemical performance of the material. Material was found to be sufficiently stable upon cycling, retaining 75% of initial capacity after 100 cycles were observed and 57% after 1000 cycles were observed. We have attempted to use 50% CH_3_CN as an electrolyte to prevent plausible washout of the SPVC, but no effect was observed, since, as was shown later, no washout occurred during the cycling of the film.

Ion transport studies showed the prevailing proton transport, which is the fastest ion transport in aqueous solutions. When we replaced the H^+^ electrolyte with the Li^+^, reversible CV distortion and capacity drop were observed, indicating the sensitivity of the material to the nature of cationic transport. Electronic conductivity limitation was excluded by the conductance measurements and the oxidative saturation experiment. Analyzing the dependences of peak current vs. the scan rate, we can conclude that they obey nearly linear relation, which indicates the kinetic limitation of the redox process. Rate constants of heterogeneous charge transfer (*k*_s_) obtained by the Laviron method were about 5 times lower compared with the PEDOT:PSS [22], which indicates that the charge transfer between the SPVC and PEDOT components limits an overall transfer. Spectroelectrochemical OCP studies indicate that the charge transfer between the PEDOT and SPVC components is within the scale of several minutes, which fits the obtained values of *k*_s_ (0.057–0.084 s^−1^). According to the literature data, the density of charge transfer pathways (in our case, there should be contacts between the PEDOT and SPVC chains) strongly affects the *k*_s_ values [36].

Most of the materials show the thickness capacity limitation phenomenon, in which the practical capacity of the film is inversely proportional to its thickness, due to the diffusion or kinetic limitations. For the PEDOT:SPVC film, the thickness limitation is very strict: the capacity does not reach the plateau even at areal loadings as low as 10.6 µg·cm^−2^ (which corresponds to the 90 nm film thickness, considering the density to be the same as with PEDOT:PSS). Further thickness reduction was impossible due to the technical difficulties of the determination of gravimetric capacity; however, the capacity shows the tendency to increase at lower film thickness. To overcome this limitation and prospectively increase the practical capacity of the PEDOT:SPVC, it was deposited on the SWCNT felt electrode. Unfortunately, the obtained capacity (49.3 mAh·g^−1^, which corresponds to the capacitance of 178 F·g^−1^ or 89 F·cm^−2^) was significantly lower than the one for the thin film. This effect may be caused due to the low diameter of SWCNT (2–3 nm), which makes the nanotube surface too curved and prevents effective contact with the rigid PEDOT chain.

Summarizing the discussion, the capacity of both thin PEDOT:SPVC film and nanocomposite electrode PEDOT:SPVC/SWCNT surpasses the previous results [22], which means that the optimization of the composition and morphology of this material was fruitful in terms of the electrochemical capacity. Future studies may be directed toward several pathways: further optimization including solvent and temperature variation, surfactant additives etc., search for an optimal support (such as multiwalled nanotubes) for the material deposition to effectively overcome the capacity limitation and utilization of the SPVC with shorter polymeric chains to increase the phase homogeneity of the material in order to avoid its slow diffusion during deposition.

## Data Availability

Not applicable.

## Supplementary Materials

The following supporting information can be downloaded at: https://www.mdpi.com/article/10.3390/nano12111917/s1, Figures S1–S4: XPS spectra of the PEDOT:SPVC samples; Figures S5–S8: CV stability series for the PEDOT:SPVC samples, Figures S9 and S10: conductance curves for the PEDOT:PSS and PEDOT:SPVC films, Figures S11 and S12: potential-dependent UV-Vis spectra.

## References

[1] Muench S., Wild A., Friebe C., Haupler B., Janoschka T., Schubert U.S. (2016). Polymer-Based Organic Batteries. Chem. Rev..

[2] Pirnat K., Casado N., Porcarelli L., Ballard N., Mecerreyes D. (2019). Synthesis of Redox Polymer Nanoparticles Based on Poly(vinyl catechols) and Their Electroactivity. Macromolecules.

[3] Zhang S., Zhao W., Li H., Xu Q. (2020). Cross-Conjugated Polycatechol Organic Cathode for Aqueous Zinc-Ion Storage. ChemSusChem.

[4] Lukyanov D.A., Apraksin R.V., Yankin A.N., Vlasov P.S., Levin O.V., Tolstopjatova E.G., Kondratiev V.V. (2019). Synthesis and electrochemical properties of poly(3,4-dihydroxystyrene) and its composites with conducting polymers. Synth. Met..

[5] Patil N., Aqil A., Ouhib F., Admassie S., Inganäs O., Jérôme C., Detrembleur C. (2017). Bioinspired Redox-Active Catechol-Bearing Polymers as Ultrarobust Organic Cathodes for Lithium Storage. Adv. Mater..

[6] Sun T., Li Z.J., Wang H.G., Bao D., Meng F.L., Zhang X.B. (2016). A Biodegradable Polydopamine-Derived Electrode Material for High-Capacity and Long-Life Lithium-Ion and Sodium-Ion Batteries. Angew. Chem. Int. Ed..

[7] Lee Y.A., Lee J., Kim D.W., Yoo C.Y., Park S.H., Yoo J.J., Kim S., Kim B., Cho W.K., Yoon H. (2017). Mussel-inspired surface functionalization of porous carbon nanosheets using polydopamine and Fe3+/tannic acid layers for high-performance electrochemical capacitors. J. Mater. Chem. A.

[8] Liu T., Lee B., Kim B.G., Lee M.J., Park J., Lee S.W. (2018). In Situ Polymerization of Dopamine on Graphene Framework for Charge Storage Applications. Small.

[9] Yue X., Liu H., Liu P. (2019). Polymer grafted on carbon nanotubes as a flexible cathode for aqueous zinc ion batteries. Chem. Commun..

[10] Vereshchagin A.A., Vlasov P.S., Konev A.S., Yang P., Grechishnikova G.A., Levin O.V. (2019). Novel highly conductive cathode material based on stable-radical organic framework and polymerized nickel complex for electrochemical energy storage devices. Electrochim. Acta.

[11] Vereshchagin A.A., Lukyanov D.A., Kulikov I.R., Panjwani N.A., Alekseeva E.A., Behrends J., Levin O.V. (2020). The Fast and the Capacious: A [Ni(Salen)]-TEMPO Redox-Conducting Polymer for Organic Batteries. Batter. Supercaps.

[12] Sterby M., Emanuelsson R., Huang X., Gogoll A., Strømme M., Sjödin M. (2017). Characterization of PEDOT-Quinone Conducting Redox Polymers for Water Based Secondary Batteries. Electrochim. Acta.

[13] Groenendaal L., Jonas F., Freitag D., Pielartzik H., Reynolds J.R. (2000). Poly(3,4-ethylenedioxythiophene) and Its Derivatives: Past, Present, and Future. Adv. Mater..

[14] Yang Y., Wang C., Ashraf S., Wallace G.G. (2013). Polypyrrole doped with redox-active poly(2-methoxyaniline-5-sulfonic acid) for lithium secondary batteries. RSC Adv..

[15] Patil N., Aqil M., Aqil A., Ouhib F., Marcilla R., Minoia A., Lazzaroni R., Jérôme C., Detrembleur C. (2018). Integration of Redox-Active Catechol Pendants into Poly(ionic liquid) for the Design of High-Performance Lithium-Ion Battery Cathodes. Chem. Mater..

[16] Son E.J., Kim J.H., Kim K., Park C.B. (2016). Quinone and its derivatives for energy harvesting and storage materials. J. Mater. Chem. A.

[17] Wu Y., Zeng R., Nan J., Shu D., Qiu Y., Chou S.L. (2017). Quinone Electrode Materials for Rechargeable Lithium/Sodium Ion Batteries. Adv. Energy Mater..

[18] Nagaraju D.H., Rebis T., Gabrielsson R., Elfwing A., Milczarek G., Inganäs O. (2014). Charge storage capacity of renewable biopolymer/conjugated polymer interpenetrating networks enhanced by electroactive dopants. Adv. Energy Mater..

[19] Ajjan F.N., Casado N., Rȩbiś T., Elfwing A., Solin N., Mecerreyes D., Inganäs O. (2016). High performance PEDOT/lignin biopolymer composites for electrochemical supercapacitors. J. Mater. Chem. A.

[20] Che C., Vagin M., Ail U., Gueskine V., Phopase J., Brooke R., Gabrielsson R., Jonsson M.P., Mak W.C., Berggren M. (2019). Twinning Lignosulfonate with a Conducting Polymer via Counter-Ion Exchange for Large-Scale Electrical Storage. Adv. Sustain. Syst..

[21] Chhin D., Padilla-Sampson L., Malenfant J., Rigaut V., Nazemi A., Schougaard S.B. (2019). Conducting Polymers Doped with Bifunctional Copolymers for Improved Organic Batteries. ACS Appl. Energy Mater..

[22] Lukyanov D.A., Vereshchagin A.A., Soloviova A.V., Grigorova O.V., Vlasov P.S., Levin O.V. (2021). Sulfonated Polycatechol Immobilized in a Conductive Polymer for Enhanced Energy Storage. ACS Appl. Energy Mater..

[23] Apraksin R.V., Volosatova Y.A., Volkov A.I., Vlasov P.S., Lukyanov D.A., Kulikov I.R., Eliseeva S.N., Levin O.V. (2021). Electrochemical synthesis and characterization of poly [Ni(CH3Osalen)] with immobilized poly(styrenesulfonate) anion dopants. Electrochim. Acta.

[24] Beletskii E.V., Volosatova Y.A., Eliseeva S.N., Levin O.V. (2019). The Effect of Electrode Potential on the Conductivity of Polymer Complexes of Nickel with Salen Ligands. Russ. J. Electrochem..

[25] Sauerbrey G. (1959). Verwendung von Schwingquarzen zur Wägung dünner Schichten und zur Mikrowägung. Z. Für. Phys..

[26] Topart P.A., Noel M.A.M. (2002). High-Frequency Impedance Analysis of Quartz Crystal Microbalances. 2. Electrochemical Deposition and Redox Switching of Conducting Polymers. Anal. Chem..

[27] Fan B., Mei X., Ouyang J. (2008). Significant Conductivity Enhancement of Conductive Poly(3,4-ethylenedioxythiophene):Poly(styrenesulfonate) Films by Adding Anionic Surfactants into Polymer Solution. Macromolecules.

[28] Elschner A., Kirchmeyer S., Lovenich W., Merker U., Reuter K. (2010). PEDOT. Principles and Applications of an Intrinsically Conductive Polymer.

[29] Laviron E. (1979). General expression of the linear potential sweep voltammogram in the case of diffusionless electrochemical systems. J. Electroanal. Chem. Interfacial Electrochem..

[30] Kaim W., Fiedler J. (2009). Spectroelectrochemistry: The best of two worlds. Chem. Soc. Rev..

[31] Gribkova O., Iakobson O., Nekrasov A., Cabanova V., Tverskoy V., Tameev A., Vannikov A. (2016). Ultraviolet-Visible-Near Infrared and Raman spectroelectrochemistry of poly(3,4-ethylenedioxythiophene) complexes with sulfonated polyelectrolytes. The role of inter- and intra-molecular interactions in polyelectrolyte. Electrochim. Acta.

[32] Victor T.A.N.Y., Alexy A., Usov A.A., Goncharova A.N., Leskin N.A., Messineva N.V., Trusova M.V. (2022). Efimkina, UV/Visible Spectra. NIST Chemistry WebBook, NIST Standard Reference Database.

[33] Patil N., Mavrandonakis A., Jérôme C., Detrembleur C., Casado N., Mecerreyes D., Palma J., Marcilla R. (2021). High-performance all-organic aqueous batteries based on a poly(imide) anode and poly(catechol) cathode. J. Mater. Chem. A.

[34] Karushev M.P., Timonov A.M. (2012). Adsorption-electrochemical modification of nanoporous carbon materials by nickel complexes with Schiff bases. Russ. J. Appl. Chem..

[35] Essafi W., Spiteri M.-N., Williams C., Boue F. (2009). Hydrophobic Polyelectrolytes in Better Polar Solvent. Structure and Chain Conformation As Seen by SAXS and SANS. Macromolecules.

[36] Wijeratne K., Ail U., Brooke R., Vagin M., Liu X., Fahlman M., Crispin X. (2018). Bulk electronic transport impacts on electron transfer at conducting polymer electrode-electrolyte interfaces. Proc. Natl. Acad. Sci. USA.

