# Supplementary material for: Optimization of Sulfonated Polycatechol:PEDOT Energy Storage Performance by the Morphology Control
Source: Nanomaterials (Basel). 2022 Jun 3;12(11):1917. doi: 10.3390/nano12111917 (PMC9182356; doi:10.3390/nano12111917)
Supplement: Supplementary file 1 [file nanomaterials-12-01917-s001.zip › nanomaterials-1720392-supplementary.pdf]

nanomaterials

Supplementary Material for:

Optimization of sulfonated polycatechol:PEDOT interpenetrating network energy storage performance by the morphology control

Anatoliy A. Vereshchagin, Vasiliy V. Potapenkov, Petr S. Vlasov, Daniil A. Lukyanov and Oleg V. Levin *

Institute of Chemistry, Saint Petersburg University, St. Petersburg 199034, Russia; anatoliy_ve@mail.ru (A.A.V.); bacuy.99@mail.ru (V.V.P.); petr_vlasov@mail.ru (P.S.V.); lda93@yandex.ru (D.A.L.)

* Correspondence: o.levin@spbu.ru; Tel.: +7-(812)-4286900

Citation: Vereshchagin, A.A.; Potapenkov, V.V.; Vlasov, P.S.; Lukyanov, D.A.; Levin, O.V. Optimization of Sulfonated Polycatechol: PEDOT Energy Storage Performance by the Morphology Control. *Nanomaterials* **2022**, *12*, 1917. https://doi.org/10.3390/nano12111917

Academic Editor: Wei Zhang

Received: 25 April 2022
Accepted: 30 May 2022
Published: 3 June 2022

Publisher's Note: MDPI stays neutral with regard to jurisdictional claims in published maps and institutional affiliations.

Copyright: © 2022 by the authors. Submitted for possible open access publication under the terms and conditions of the Creative Commons Attribution (CC BY) license (https://creativecommons.org/licenses/by/4.0/).

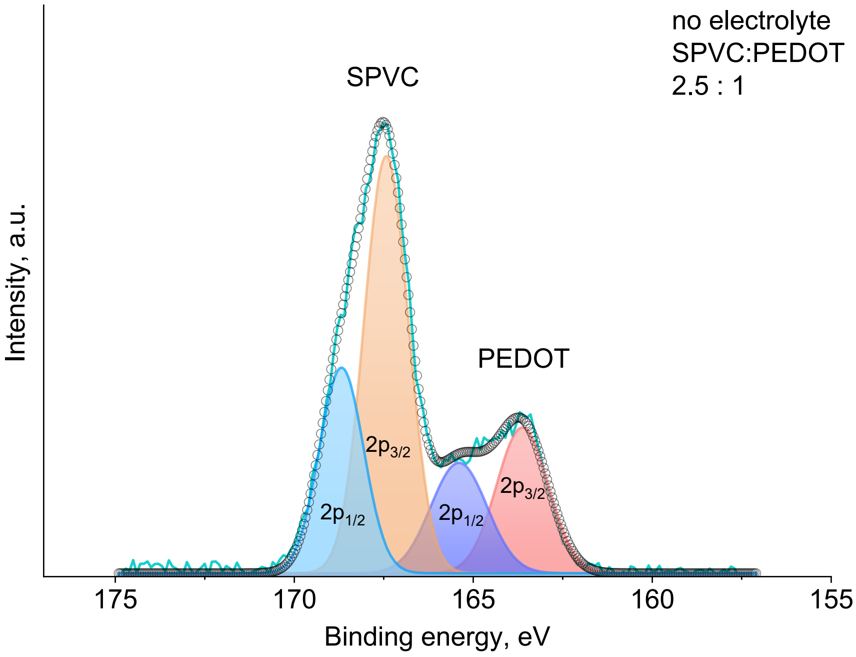

Figure S1. Fitted XPS spectrum of the PEDOT:SPVC film, no $NaClO_4$.

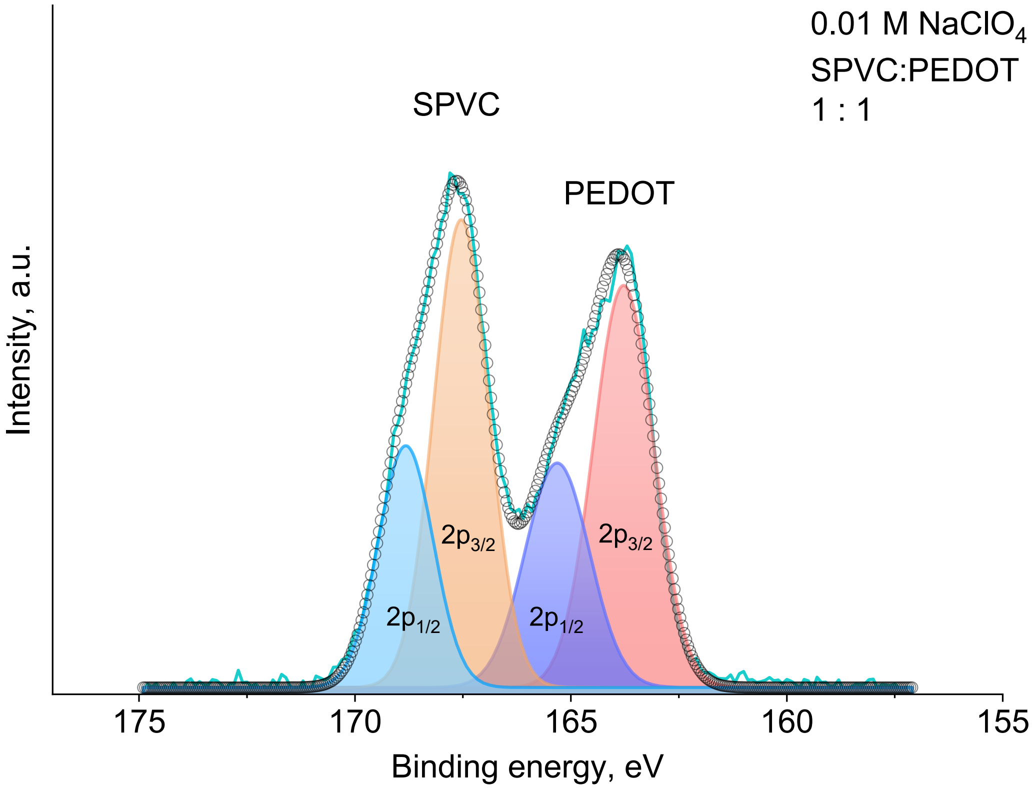

Figure S2. Fitted XPS spectrum of the PEDOT:SPVC film, 0.01 M $NaClO_4$.

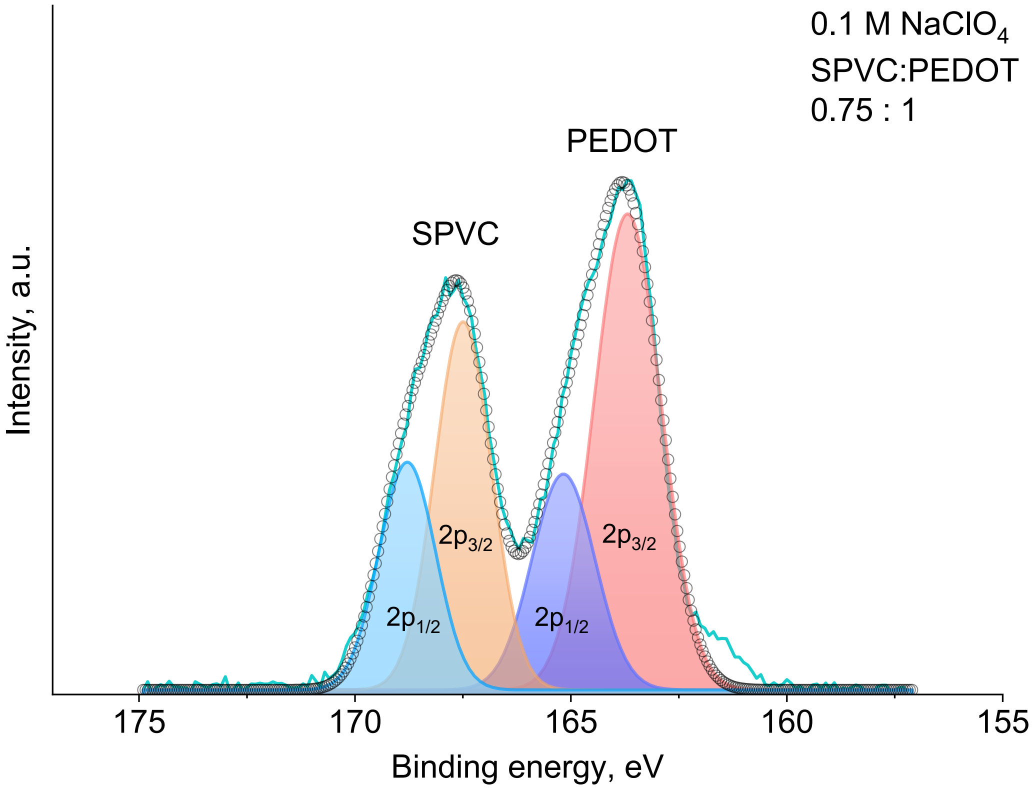

Figure S3. Fitted XPS spectrum of the PEDOT:SPVC film, 0.1 M $NaClO_4$.

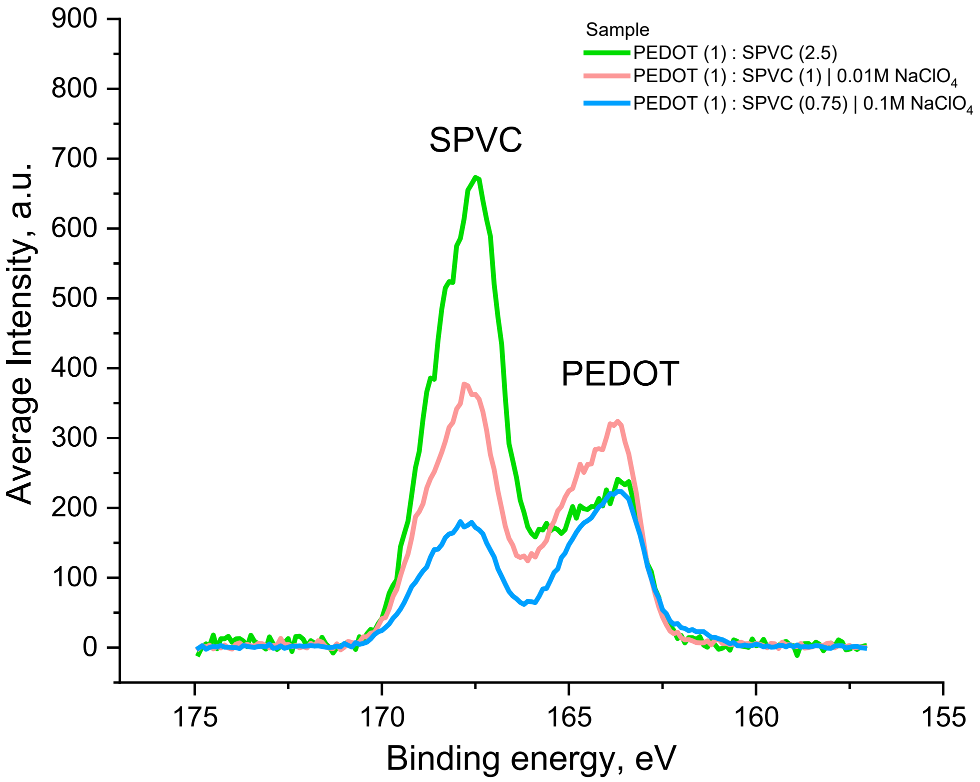

Figure S4. Overlayed XPS spectra of the PEDOT:SPVC with $NaClO_4$ additives.

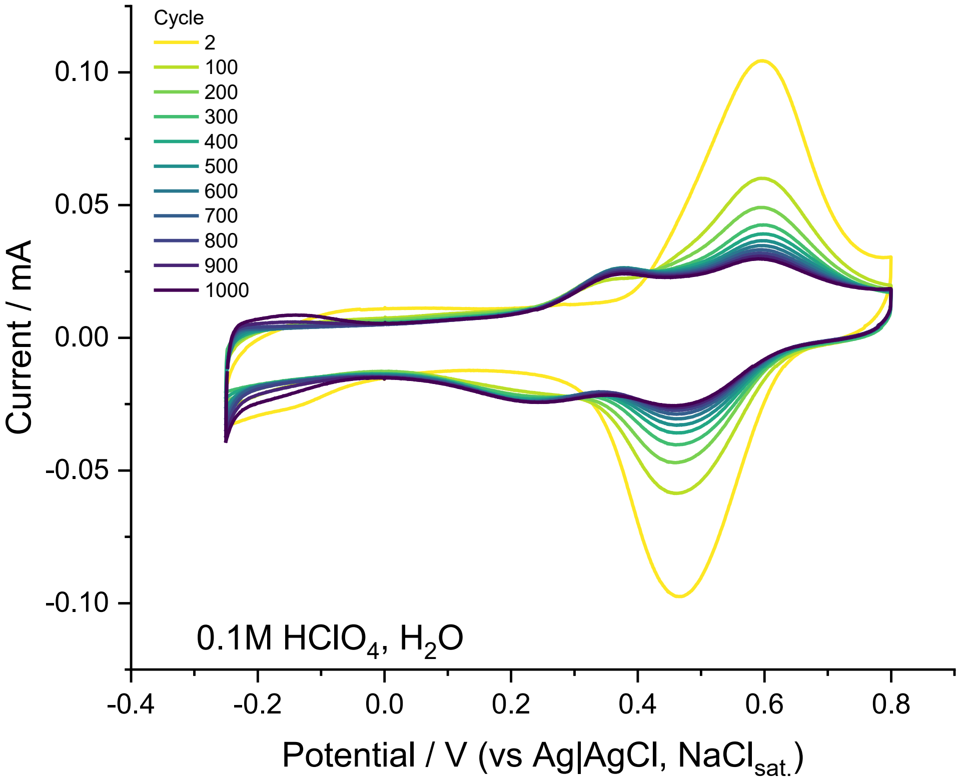

Figure S5. CV stability of PEDOT:SPVC, 20 mV s^{-1}, 0.1 M $HClO_4$.

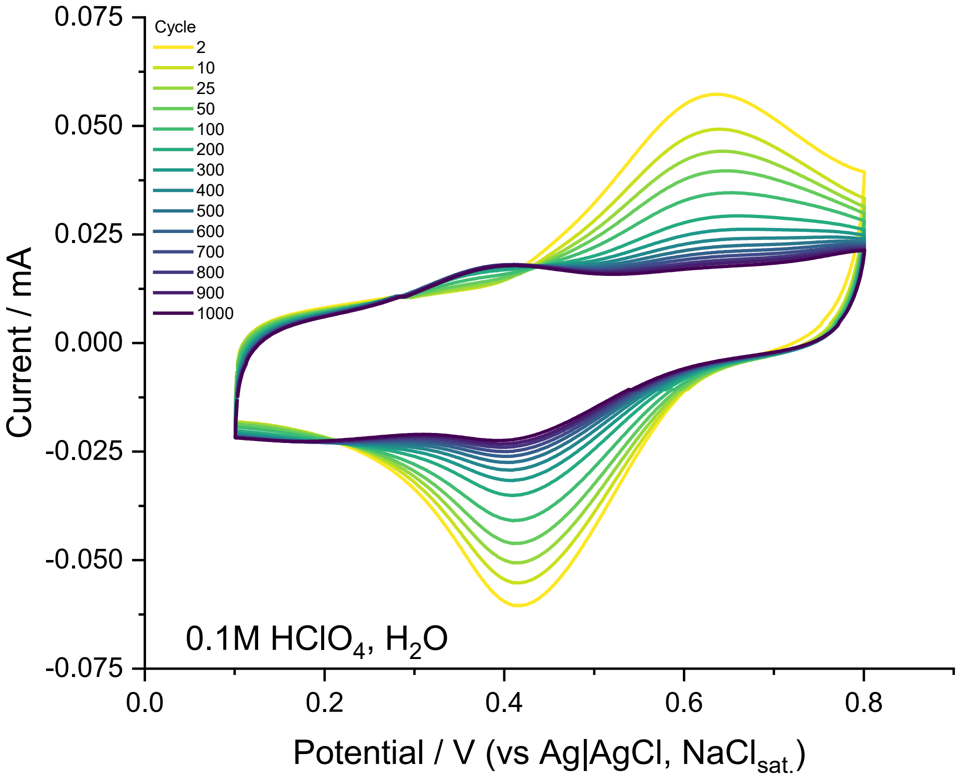

Figure S6. CV stability of PEDOT:SPVC, short range, 20 mV s^{-1}, 0.1 M $HClO_4$.

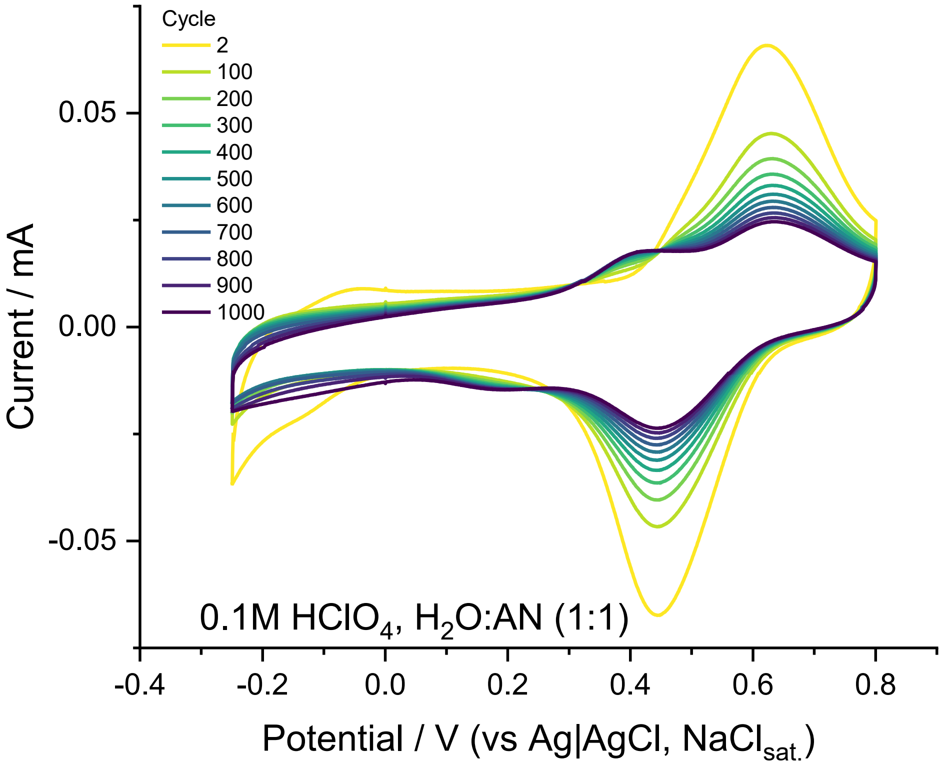

Figure S7. CV stability of PEDOT:SPVC, CH_3CN additive, 20 mV s^{-1}, 0.1 M $HClO_4$.

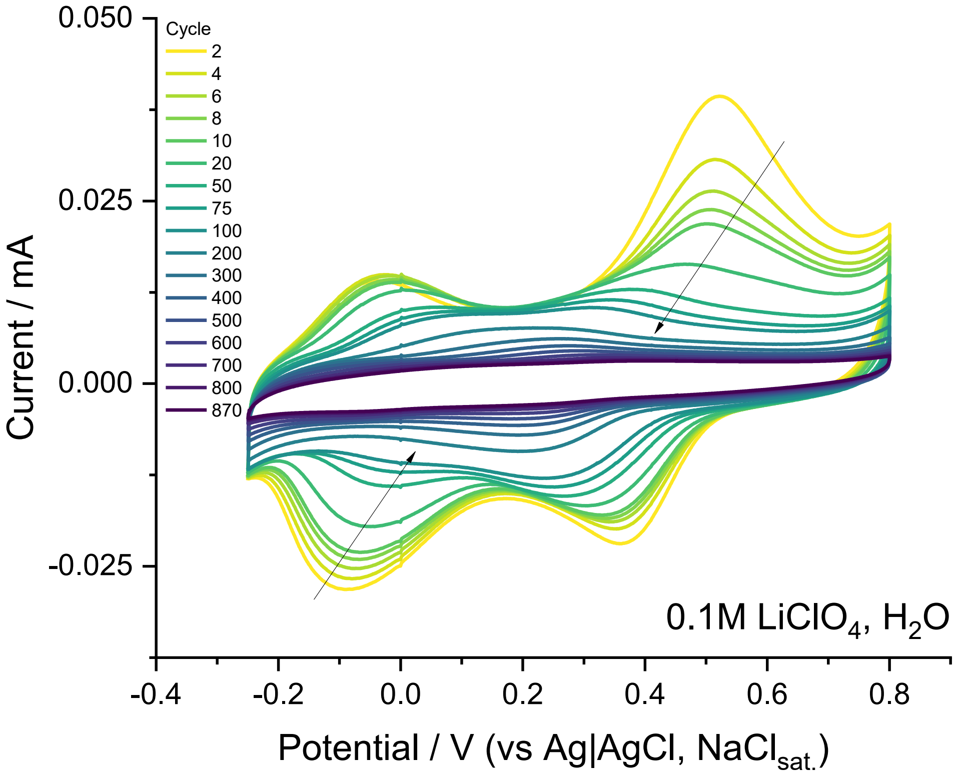

Figure S8. CV stability of PEDOT:SPVC, 20 mV s^{-1}, 0.1 M $LiClO_4$.

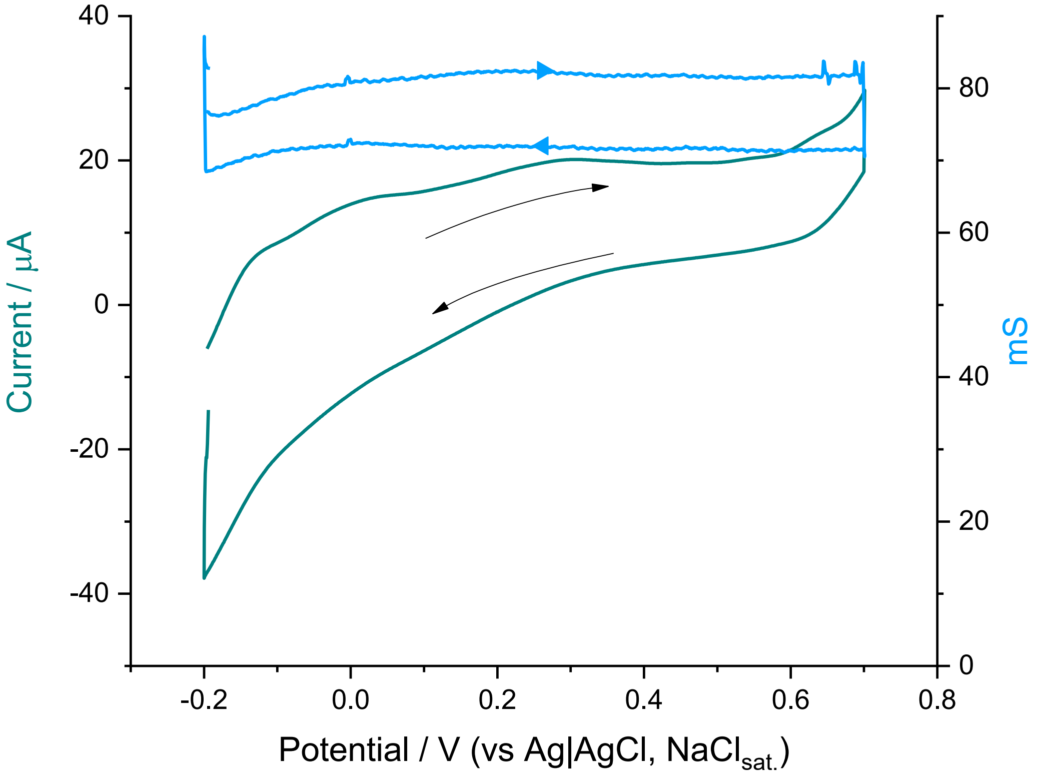

Figure S9. Conductance of the PEDOT:PSS film, 5 mV s^{-1}, 0.1 M $HClO_4$.

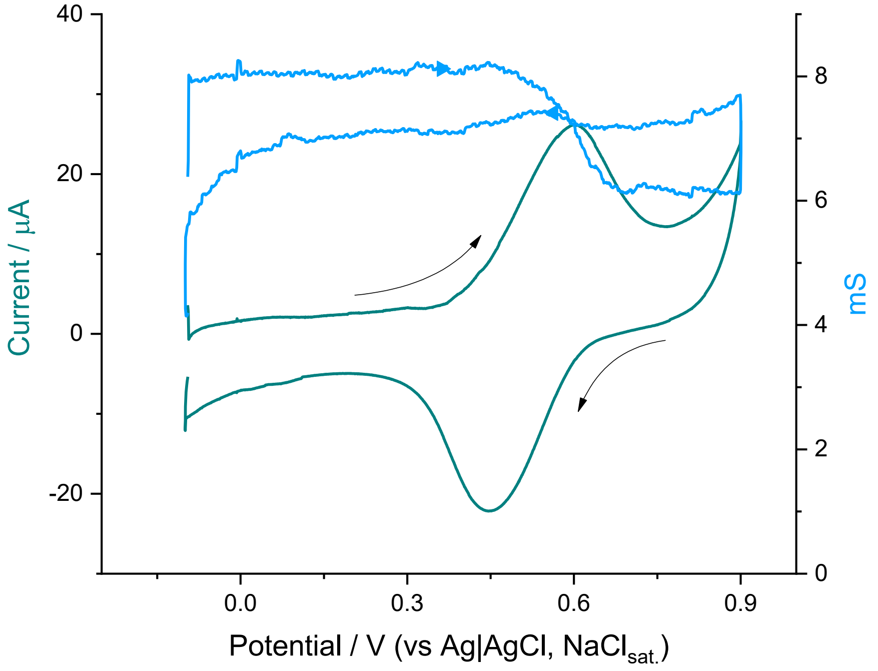

Figure S10. Conductance of the PEDOT:SPVC film, 5 mV s-1, 0.1 M $HClO_4$.

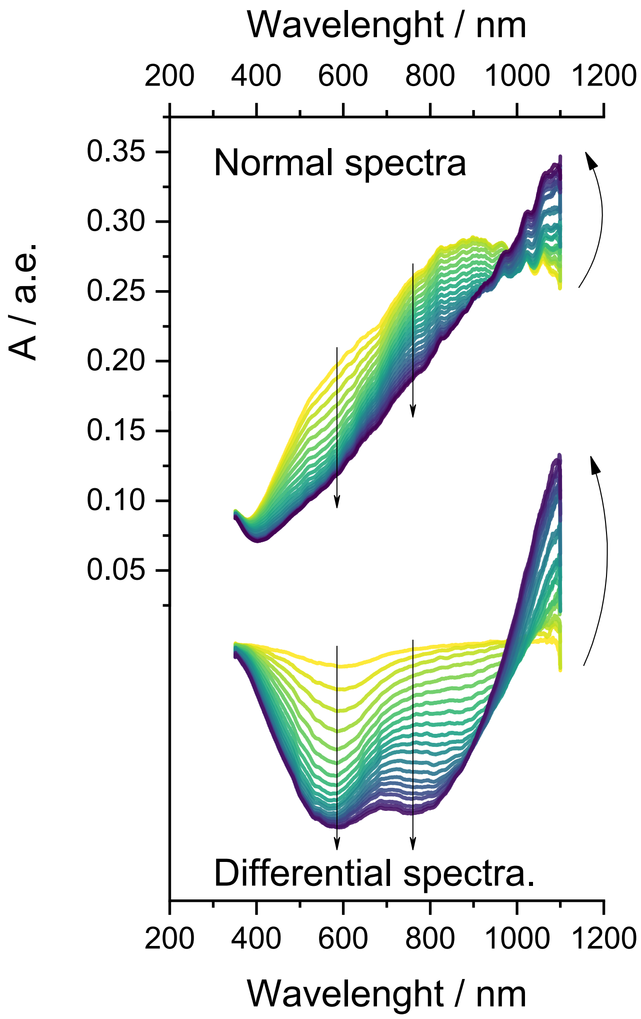

Figure S11. Potential-dependent UV-Vis spectra of PEDOT:PSS, 0.1 M $HClO_4$.

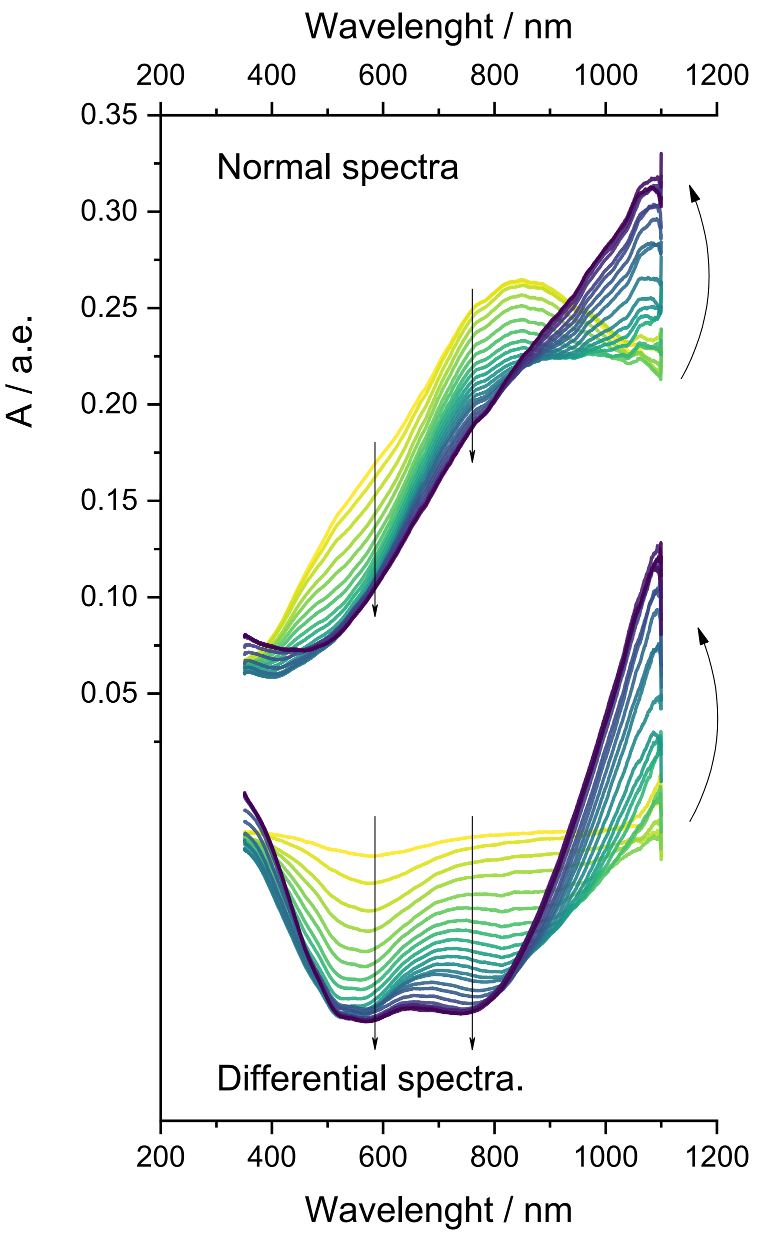

Figure S12. Potential-dependent UV-Vis spectra of PEDOT:SPVC, 0.1 M $HClO_4$.
